# Phytol and Heptacosane Are Possible Tools to Overcome Multidrug Resistance in an In Vitro Model of Acute Myeloid Leukemia

**DOI:** 10.3390/ph15030356

**Published:** 2022-03-15

**Authors:** Manuela Labbozzetta, Paola Poma, Marco Tutone, James A. McCubrey, Maurizio Sajeva, Monica Notarbartolo

**Affiliations:** 1 Department of Biological, Chemical and Pharmaceutical Science and Technology (STEBICEF), University of Palermo, 90128 Palermo, Italy; manuela.labbozzetta@unipa.it (M.L.); paola.poma@unipa.it (P.P.); marco.tutone@unipa.it (M.T.); maurizio.sajeva@unipa.it (M.S.); 2 Department of Microbiology and Immunology, Brody School of Medicine at East Carolina University, Greenville, NC 27858, USA; mccubreyj@ecu.edu

**Keywords:** P-glycoprotein, multidrug resistance, acute myeloid leukemia cell, P-gp inhibitors, phytol, heptacosane

## Abstract

Drug resistance is the ability of cancer cells to gain resistance to both conventional and novel chemotherapy agents, and remains a major problem in cancer therapy. Resistance mechanisms are multifactorial and involve more strictly pharmacological factors, such as P-glycoprotein (P-gp) and biological factors such as inhibitor of apoptosis proteins (IAPs) and the nuclear factor-kappa B (NF-κB) pathway. Possible therapeutic strategies for the treatment of acute myeloid leukemia (AML) have increased in recent years; however, drug resistance remains a problem for most pa-tients. Phytol and heptacosane are the major compounds of *Euphorbia intisy* essential oil (EO) which were demonstrated to inhibit P-gp in a multidrug resistant in vitro model of AML. This study investigated the mechanism by which phytol and heptacosane improve P-gp-mediated drug transport. Phytol suppresses the P-gp expression via NF-κB inhibition and does not seem to act on the efflux system. Heptacosane acts as a substrate and potent P-gp inhibitor, demonstrating the ability to retain the substrate doxorubicin inside the cell and enhancing its cytotoxic effects. Our results suggest that these compounds act as non-toxic modulators of P-gp through different mechanisms and are able to revert P-gp-mediated drug resistance in tumor cells.

## 1. Introduction

Resistance to chemotherapy is a major public health problem. Multidrug resistance and subsequent treatment ineffectiveness are known to be responsible for 90% of cancer-related deaths. Moreover, when a cancer cell acquires the resistant phenotype, it also becomes resistant to anticancer chemotherapy drugs that are structurally and functionally different from the drug that generated the resistance. In the last decade, target cancer therapies have been developed in the hope of improving anticancer efficacy. While many of the targeted therapy drugs showed promising early clinical outcomes with improved overall survival, a large number of the patients receiving targeted therapy developed drug resistance.

Overexpression of members of the adenosine triphosphate binding cassette (ABC) transporters, including P-glycoprotein (MDR1 or ABCB1), the multidrug resistance associated protein (MRP1 or ABCC1) and the breast cancer resistance protein (BCRP1 or ABCG2), are implied in the genesis of multidrug resistance (MDR) in various tumor models [1].

In this context, P-gp, also known as multidrug resistance protein 1, is an efflux pump playing a key role in regulating the intake of anticancer chemotherapy drugs responsible for multidrug resistance in various types of cancer [2,3,4]. Numerous pre-clinical and clinical studies have investigated the possibility of using P-gp inhibitors for the treatment of cancer with the idea of enhancing chemotherapy efficacy [5,6,7]. More recently, some evidences have demonstrated a P-gp reversing activity by using natural products as flavonoids, curcuminoids, taccalonolides, and terpenes [8,9]. In this context, the development of natural P-gp transporter inhibitors that are more potent and less toxic is underway. These inhibitors are divided into two subclasses. Inhibitors of the first-class transport themselves into cells and then they inhibit the function of transporters as competitive antagonists. Inhibitors of the second-class are not transported into cells but affect transporter function [9]. In general, natural product modulators of multidrug resistance (MDR) are substrates for transporters like P-gp and compete with anticancer agents for binding to the active site of transporters (DBP, drug binding pocket) and reducing drug efflux. Most of these natural compounds have the capability to modulate the outflow, inhibit ATPase activity, and reduce the expression of the gene that encodes for P-gp at the same time. A large number of natural products were re-ported to be P-gp inhibitors by blocking the ATPase activity through nucleotide binding pocket or ATP binding pocket (NBP) which is their major binding site [9,10]. For these reasons, these compounds are considered to be fourth-generation inhibitors and their use is a promising alternative to overcome the numerous side effects and the drug resistance associated with anticancer drugs. Some of these compounds have antitumor activities on different types of tumors, all characterized by scarce responsiveness to chemotherapeutic drugs with an MDR phenotype and inhibition of cell growth and an induction of cell death [10,11,12,13,14,15,16,17,18,19,20], due to the inhibition of the nuclear transcription factor NF-κB pathway [21,22,23].

AML is the most common acute leukemia in adults. It is a very heterogeneous malignancy and even if the therapeutic choice is wide (combination of antiblastic and biotech drugs with allogeneic stem cell transplantation for eligible patients), the vast majority of patients develop in very short time multidrug resistance that makes urgent the research of molecules able to overcome this condition [24,25]. In the refractory or resistant forms some target therapies are possible but depend on the genetic mutations found in leukemia cells [26,27]. Unfavorable prognostic factors in AML such as the overexpression of NF-κB and IAPs also in correlation with the phenomenon of MDR have been identified and P-gp is a potential therapeutic target because the most clinical resistant tumors are characterized by its overexpression [28,29]). Poma et al., [30] showed that *E. intisy* EO acts as modulators of P-gp in an in vitro model of AML multidrug resistant HL-60R. In the present paper we wanted to verify if the activity of *E. intisy* EO was due to its major compounds, heptacosane and phytol. Heptacosane is a straight-chain alkane with 27 carbon atoms. It may be a component of petroleum products but it is naturally present in several plants and insects and also found in the tissues of ruminants [31]. Phytol is a ditherpene widely distributed in plants. Several biomedical activities have been described [32]. We investigated the effects of phytol and heptacosane, identifying different mechanisms of action through which the molecules act as modulators of P-gp in a model of AML multi-drug resistant HL-60R.

## 2. Results

### 2.1. In Vitro Cytotoxicity Effects of Phytol and Heptacosane

Our first evaluation concerned the cytotoxic activity of the two major components of the *E. intisy* EO phytol and heptacosane, on two cell lines, HL-60 and its multidrug resistant variant HL-60R, used as tumor model, on hTERT RPE-1 immortalized retinal pigment epithelial cell line, 1-7HB2 human mammary luminal epithelial cell line. All cell lines were treated with a large range of concentrations of phytol and heptacosane for 72 h. Both molecules did not induce cell growth inhibition up to the concentration of 100 µg/mL, in tumor cells and in normal cell line (Table 1). These data indicate that the two major compounds of the EO were not responsible alone for the inhibitory effect on cell growth and the block in the preG_0_-G_1_ position of the cell cycle in both cell lines, that was observed when the whole EO is used [30].

### 2.2. Effects on the Activation of the NF-κB Signaling Pathway

**Figure 1:**
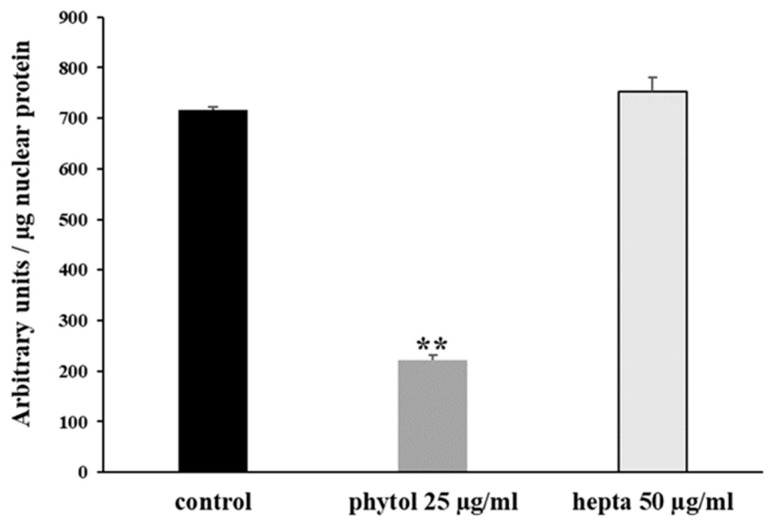
NF-κB (p65 subunit) DNA binding capacity in nuclear extracts of HL-60R cells. The cells were treated for 24 h with phytol and heptacosane (hepta) at the indicated concentrations. Results (mean ± standard error of three experiments carried out in duplicate) are expressed as arbitrary units/µg protein of cells nuclear extracts. Statistical differences are ** *p* < 0.01, vs. the control (one-way ANOVA followed by Tukey’s test).

Previous data indicated the strong reduction of the constitutive activation of the NF-κB pathway caused by different essential oils, characterized by high phytol content. We evaluated this effect after 24 h of treatment with phytol (25 µg/mL) and heptacosane (50 µg/mL) in the HL-60R cell line. These two concentrations are in accordance with the percentage of the compounds in the *E. intisy* EO. The results indicate that only phytol causes a strong reduction of NF-κB DNA-binding activity in HL-60R (Figure 1).

**Figure 2:**
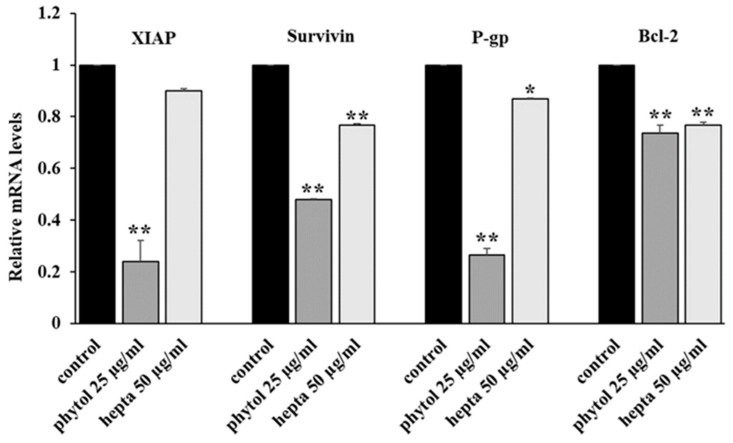
Genes mRNA expression levels by quantitative polymerase chain reaction in HL-60R cells. The cells were treated for 24 h with phytol (25 μg/mL) or heptacosane (hepta; 50 μg/mL). Data are expressed as mean ± standard error of three experiments. Differences when treatments are compared to the control, ** *p* < 0.01, * *p* < 0.05 (one-way ANOVA followed by Tukey’s test).

**Figure 3:**
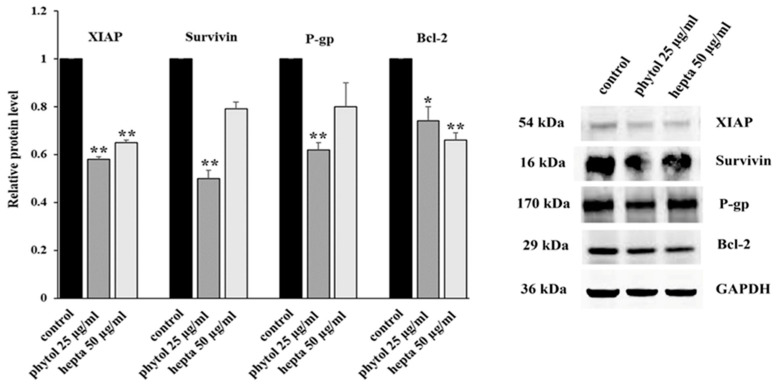
Western blotting analysis of the levels of XIAP, survivin, P-gp and Bcl-2 in HL-60R cells. The cells were treated for 24 h with phytol (25 μg/mL) or heptacosane (hepta; 50 μg/mL). On the left the results expressed as relative protein level (mean ± standard error of three experiments); differences when treatments are compared to the control, ** *p* < 0.01, * *p* < 0.05 (one-way ANOVA followed by Tukey’s test). On the right, the results of a representative experiment. Irrelevant parts of the gel image (such as blank lanes and lanes with molecular weight markers) are deleted. The samples were derived from the same experiment and that gels/blots were processed in parallel.

We evaluated the effects of phytol and heptacosane on the expression, both at mRNA and protein level, of some NF-κB target genes, like some antiapoptotic factors (survivin, XIAP, and Bcl-2), and the multidrug transporter P-gp. Phytol produces a significant decrease both in mRNA and protein levels of the targets, while heptacosane has a lower inhibiting action (Figure 2 and Figure 3).

### 2.3. Binding Site Detection

**Figure 4:**
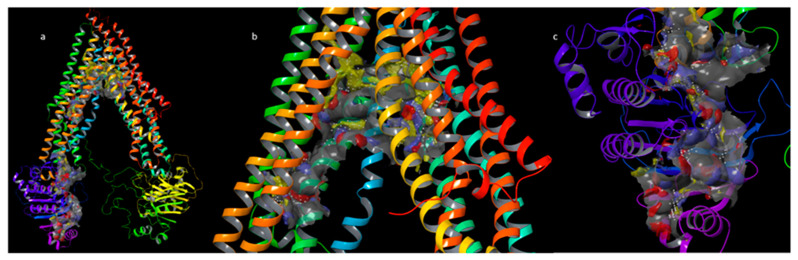
(**a**) DBP and NBP retrieved by SiteMap analysis; (**b**) point of view of DBP; (**c**) point of view of NBP. The phobic surfaces are reported in yellow, the HB acceptor surfaces are reported in red, HB donor surfaces are reported in blue, the surfaces of the two pockets are reported in grey.

In order to gain further insights into the protein hot spots involved in the binding sites, all of the potential druggable sites of the P-gp were explored starting from the homology model of the P-gp previously built (Supplementary Material and Figure S1). For this purpose, we performed the binding site detection by using SiteMap [33]. As reported [34], the P-gp has two principal binding sites: the DBP, and the NBP or ATP binding pocket. Despite the identification of several P-gp modulators, the real mechanism of action of these modulators should be better clarified. Some modulators seem to act through competitive interactions with the substrate over the DBP [35], while others could allosterically interact with different sites [36]. Some modulators interact with the NBP causing an inhibition of the ATPase activity. SiteMap reported two putative binding sites which are ascribable to the known binding pockets. In the 1980s, the discovery by Tsuruo and colleagues [37] that verapamil can inhibit P-gp has marked a major milestone in this field of research. In previous papers, it was described that verapamil could bind the DBP and the NBP [34,38,39]. For these reasons, we focused our investigation on both DBP and NBP. The retrieved pocket for the DBP showed the higher Site score value = 1.145 and DScore (Druggability Score) value = 1.245 concerning the retrieved pockets for NBP. This last score is one of the keys for distinguishing “difficult” and “undruggable” targets from “druggable” ones [32]. In particular, the identified DBP is constituted by 385 site points. As a rough rule of thumb, 2 to 3 site points typically correspond to each atom of the bound ligand, including hydrogens. The size of the site is often a good indicator of the preferred binding site. The volume is 1549 Å^3^, and in terms of hydrophobic and hydrophilic characters of the site, the identified pocket has a prevalent phobic balance as can be noted in Figure 4b with a presence of large yellow surfaces showing the phobic surface of the pocket. The retrieved pocket for the NBP showed the Site score value = 0.993 and DScore value = 1.056. In particular, the identified NBP is constituted by 210 site points. The size of the site is often a good indicator of the preferred binding site. The volume is 586 Å^3^, and in terms of the hydrophobic and hydrophilic characters of the site, there is a slight hydrophilic balance (Figure 4c).

### 2.4. Docking and Binding Free Energy Calculation

**Figure 5:**
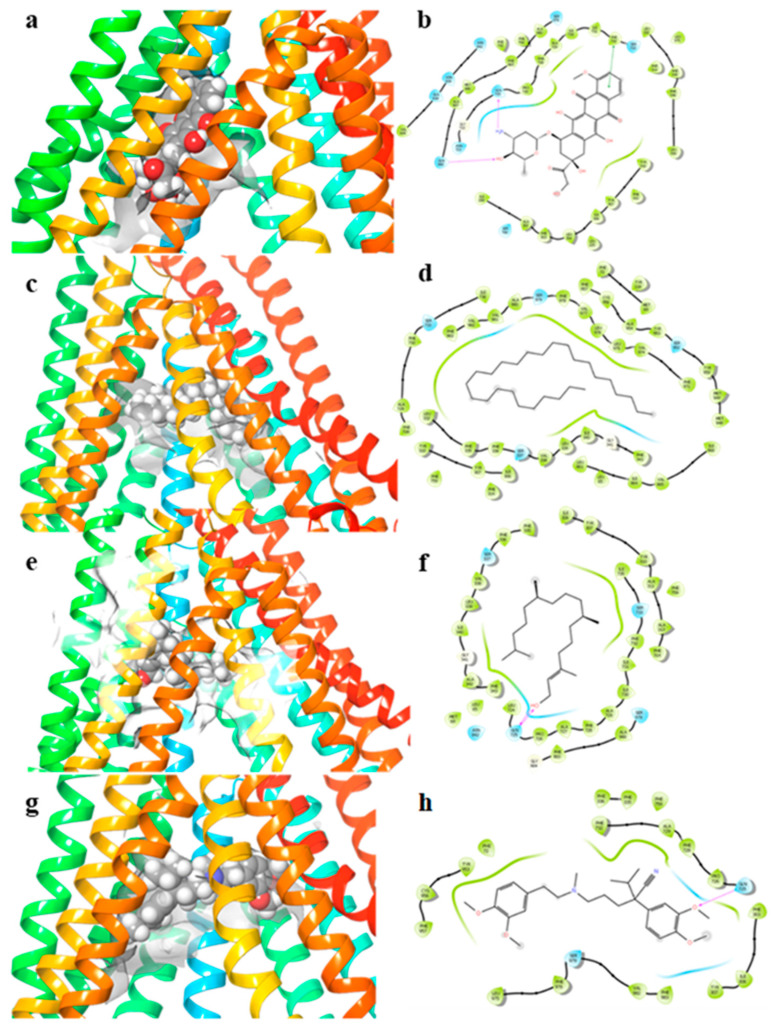
3D docking poses in the DBP and 2D ligand–protein interaction diagrams. (**a**,**b**) doxorubicin; (**c**,**d**) heptacosane; (**e**,**f**) phytol; (**g**,**h**) verapamil. The purple arrows in the 2D ligand–protein interaction diagrams represent the H-bond interaction. The green line in the 2D ligand–protein interaction diagrams represent the pi-stacking interactions.

**Figure 6:**
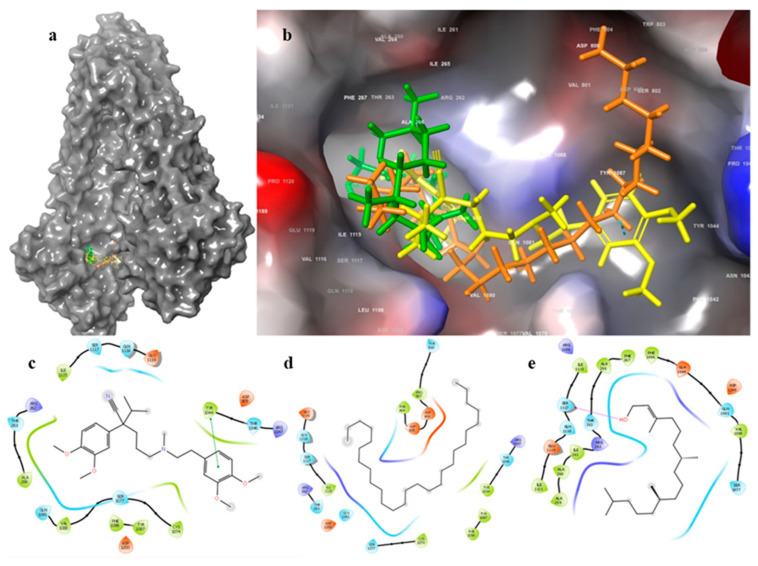
(**a**) Docking poses of verapamil, heptacosane and phytol in the NBP, (**b**) Zoom in of verapamil, heptacosane and phytol in the NBP. 2D ligand–protein interaction diagrams: (**c**) verapamil; (**d**) heptacosane; (**e**) phytol. The purple arrows in the 2D ligand–protein interaction diagrams represent the H-bond interaction. The green lines in the 2D ligand–protein interaction diagrams represent the pi-stacking interactions.

To study the binding mode of our compounds, phytol and heptacosane, we used the Glide XP protocol as previously reported [40]. MM-GBSA calculation followed docking for the determination of the binding free energy of each compound with P-gp. The binding mode of our compounds has been compared with the P-gp known inhibitor verapamil, and the P-gp substrate doxorubicin in the DBP. Docking of doxorubicin (∆G = −52.1 kcal/mol) to P-gp showed a lower binding free energy than phytol (∆G = −75.8 kcal/mol), heptacosane (∆G = −114.2 kcal/mol) and verapamil (∆G = −82.7 kcal/mol). All of the three inhibitors assume a pose along the floor of the DBP while doxorubicin has a preference with the wall of the DBP, interacting with an H-bond with Gln725. Except for heptacosane, which shows exclusively phobic interaction along only 3 Å of diameter. Doxorubicin showed a pi-stacking interaction with Phe732 and the methoxyphenyl ring, and an additional H-bond with Gln990. The major part of the interactions of phytol are with hydrophobic residues. The output of docking for the three inhibitors in the NBP showed that verapamil has the higher binding free energy (∆G = −42.3 kcal/mol). Heptacosane showed a slightly higher binding free energy than phytol (∆G = −30.2 kcal/mol and ∆G = −29.7 kcal/mol, respectively). Verapamil and heptacosane showed a similar binding mode occupying the deep hydrophobic subpocket (Table 2, Figure 5 and Figure 6).

### 2.5. Effects of Phytol and Heptacosane on Intracellular Accumulation of Doxorubicin in the HL-60R Cell Line

**Figure 7:**
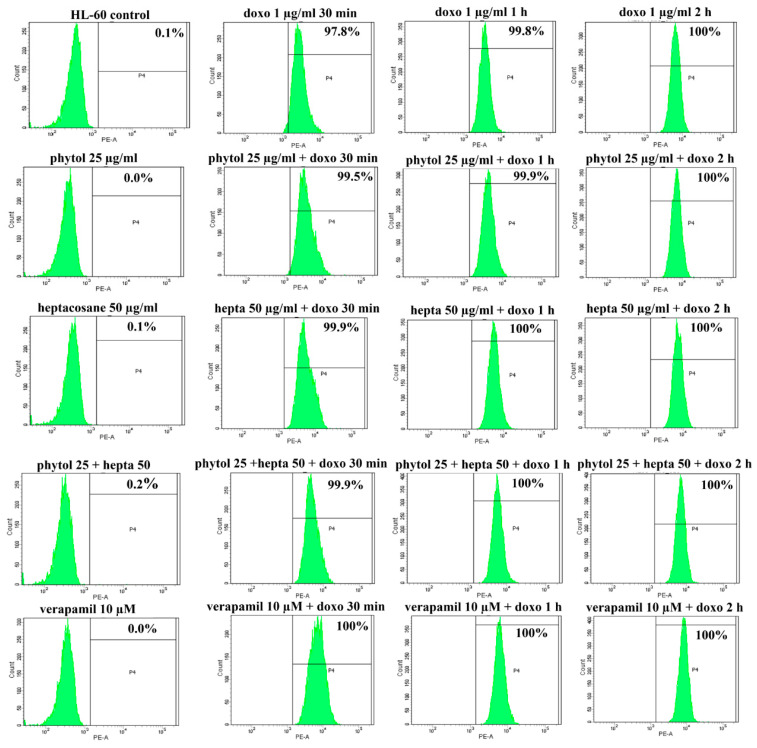
Effects of phytol and heptacosane on intracellular accumulation of doxorubicin in HL-60 cell line. Representative example of flow cytometry analysis of intracellular accumulation of doxorubicin (doxo; 1 µg/mL) after different times of incubation (30 min, 1 h and 2 h) in HL-60 cells pre-treated with phytol (25 µg/mL), heptacosane (hepta; 50 µg/mL), alone and in combination, or verapamil 10 µM for 24 h.

**Figure 8:**
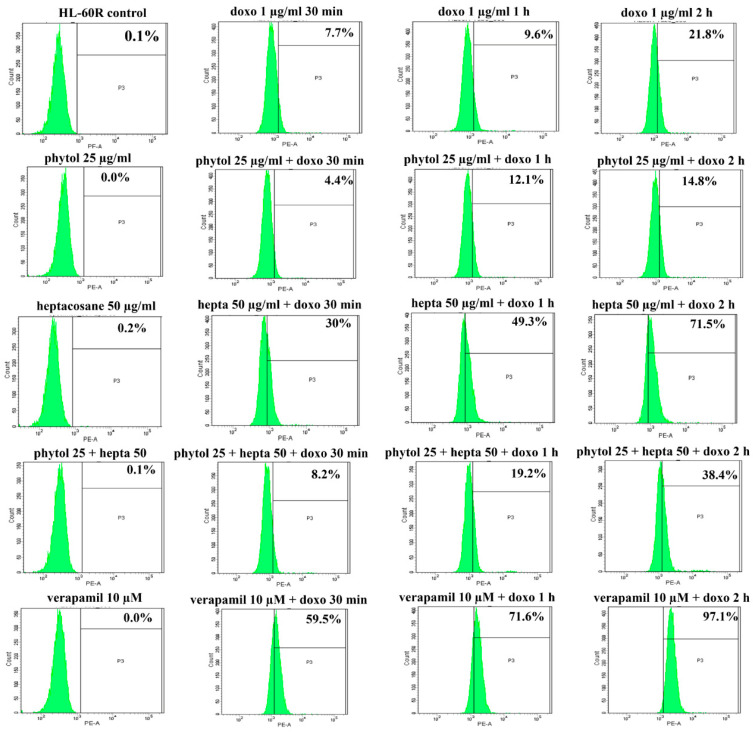
Effects of phytol and heptacosane on intracellular accumulation of doxorubicin in HL-60R cell line. Representative example of flow cytometry analysis of intracellular accumulation of doxorubicin (doxo; 1 µg/mL) after different times of incubation (30 min, 1 h and 2 h) in HL-60R cells pre-treated with phytol (25 µg/mL), heptacosane (hepta; 50 µg/mL), alone and in combination, or verapamil 10 µM for 24 h.

The docking data suggest a role of phytol and heptacosane in blocking the efflux function of P-gp. Based on these results, we wanted to investigate whether phytol and heptacosane were able to cause inhibition of the P-gp pump, through the accumulation assay of its substrate doxorubicin. HL-60 and HL-60R cell lines were pretreated for 24 h with phytol (25 µg/mL), heptacosane (50 µg/mL) alone or in combinations with both. After this incubation, 1 µg/mL of doxorubicin was added at 30 min, 1 h and 2 h. Data are presented as percentage of fluorescence intensity in HL-60 and HL-60R cells, measured by flow cytometric analysis (Figure 7 and Figure 8 and Table 3). The results indicate a significant increase in fluorescence in HL-60R cells only after pretreatment with heptacosane, showing an intracellular accumulation of doxorubicin after exposure at different times. Pretreating with the combination of the two compounds caused only a minimal increase in the accumulation of doxorubicin in respect to the value obtained with heptacosane alone, suggesting a competition mechanism between the two compounds. In the HL-60 cell line, pretreatment with phytol, heptacosane or their combination did not cause accumulation of doxorubicin compared to its control, confirming a selective mechanism of heptacosane on P-gp which is overexpressed only in the MDR cell line. These results suggest that in HL-60R cell line heptacosane, acts like an inhibitor of P-gp function, as verapamil that produced similar results on doxorubicin accumulation. On the contrary, phytol does not seem to act on the efflux system modulating only the expression of P-gp through the inhibition of NF-κB.

### 2.6. Cytotoxic Effects of Heptacosane in Combination with Doxorubicin

Since heptacosane increased the accumulation of doxorubicin in HL-60R we wanted to verify whether the increased intracellular accumulation of doxorubicin would lead to greater cytotoxic effects. The inhibition of cell growth was evaluated by trypan blue dye exclusion test. In Table 4, we report the percentages of cell growth inhibition by heptacosane and doxorubicin treatments. Doxorubicin inhibition of HL-60R cell growth was significantly increased by the co-treatment with heptacosane. The percentages of cell growth inhibition obtained by co-treatments versus percentages expected showed a considerable enhancement of cell growth inhibition due to doxorubicin. Therefore, we hypothesized that heptacosane may represent an effective and non-toxic inhibitor of P-gp to reverse P-gp-mediated multidrug resistance and improve the potency of anticancer drugs.

### 2.7. Effects of Phytol and Heptacosane on P-gp Activity

**Figure 9:**
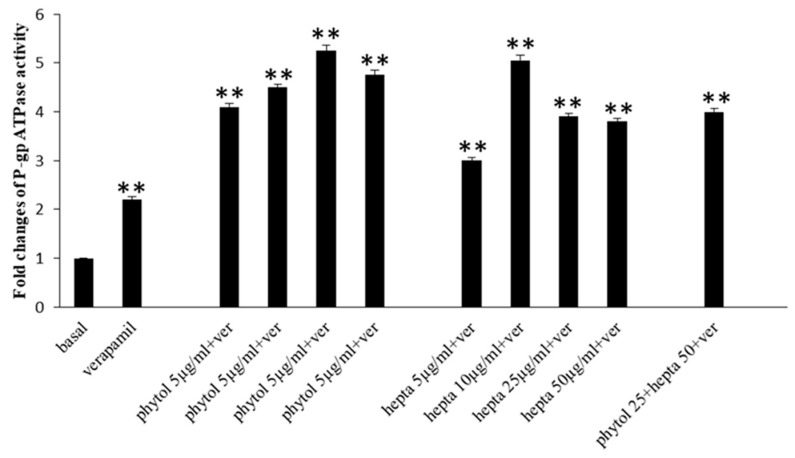
Effects of phytol and heptacosane (hepta; 5–50 µg/mL) on 0.5 mM verapamil(ver)-stimulated P-gp ATPase activity. The data are expressed as fold changes of P-gp ATPase activity compared to basal one (ΔRLU_TC_/ΔRLU_basal_) and are presented as mean ± S.E. of three experiments, each in duplicate. Differences when treatments are compared to the basal activity, ** *p* < 0.01 (one-way ANOVA followed by Tukey’s test).

To study the possible effects of phytol and heptacosane on the NBP site suggested by the docking prediction, we evaluated P-gp ATPase activity by the P-gp-Glo™ Assay that detects a luminescent signal inversely proportional to the ATP consumption. Verapamil is a substrate for transport by P-gp and has a stimulatory effect on P-gp ATPase activity. As shown in Figure 9 verapamil increased P-gp ATPase activity compared to the basal level. Both phytol and heptacosane increased verapamil-stimulated P-gp ATPase activity, probably acting as substrates for transport by P-gp and stimulators of ATP-dependent drug efflux transporter. When used together phytol and heptacosane increase the P-gp ATPase activity. The activation effect of phytol, heptacosane alone or in combination is more potent than verapamil.

## 3. Discussion

Anticancer chemotherapy shows a decrease in efficacy due to the overexpression of efflux pumps on the surface of cancer cells. This is one of the main mechanisms for re-sistance to chemotherapy and which rapidly leads to cancer progression [25]. Numerous preclinical and clinical studies have investigated the possibility of using P-gp inhibitors for the treatment of cancer with the idea of enhancing chemotherapy efficacy [7]. Verapamil, a phenylalkylamines that blocks voltage-dependent L-type calcium channels used in the treatment of hypertension and in some forms of arrhythmia, has been considered for a long time as a potent first-generation inhibitor of P-gp, but it showed high toxicity. Second-generation inhibitors of P-gp like Dexverapamil, the R-form of verapamil, have shown reduced cardiotoxicity, but little benefit. The poor clinical efficacy coupled with a low therapeutic window of these P-gp inhibitors has led to the development of more potent and selective small molecules, third-generation inhibitors such as zosuquidar, elaquidar, laniquidar, and tariquidar, which act as ATPase inhibitors with a high affinity to P-gp at nanomolar concentrations. Unfortunately, the co-administration of these third-generation inhibitors, did not improve the activity of anticancer drugs in clinical trials and none of them has been approved for therapy [41,42]. There is a growing interest in exploring the use of natural components of plants as P-gp modulators. Some natural products extracted from plants, have been studied for their modulatory properties on P-gp in the form of alkaloids, flavonoids and phenolics, coumarins, resins, peptides, saponins, terpenoids, and miscellaneous other chemical species [8,43,44]. In vitro evidence has shown that some natural products, such as curcuminoids, ursolic and oleanolic acid, and essential oils, can counteract the phenomena of drug resistance in tumor models of innate and acquired resistance such as triple negative breast cancer, hepatocellular carcinoma, and acute myeloid leukemia [44,45,46,47,48,49,50,51,52]. In this context, a recent paper has shown how essential oils are able to bypass multidrug resistance through different mechanisms all converging towards the inhibition of P-gp. In the present paper, we demonstrate the mechanism of action of two compounds, phytol and heptacosane present in the *E. intisy* EO capable of modulating drug resistance in an MDR model of acute myeloid leukemia. Unfavorable factors in AML, such as the overexpression of NF-κB and IAPs also in correlation with the phenomenon of multidrug resistance, have been identified [47,53,54]. Previous results suggest that *E. intisy* EO in the HL-60R cells reduces P-gp expression through modulation of NF-κB, caused the inhibition of its efflux function and also acts as a substrate for P-gp transport stimulating its ATPase activity which results in intracellular accumulation of doxorubicin [22]. Preliminarily, we verified that both phytol and heptacosane do not have cytotoxic effect (Table 1). Phytol acts as a strong inhibitor of the NF-κB pathway by mod-ulating its molecular targets involved in the phenomenon of resistance to apoptosis and in multidrug resistance. On the contrary, heptacosane does not interfere with NF-κB pathway but reduces same target genes probably through the modulation of other key factors [43,55] (Figure 1, Figure 2 and Figure 3). The docking study of the possible binding modes of phytol and heptacosane with the P-gp indicated that the two compounds bind the DBP site in a similar way to verapamil. Heptacosane appears to be the one with the most favorable interaction, assuming a conformation that could close the efflux pump, moreover, the partial overlap on the binding site between phytol and heptacosane could explain the competition phenomena subsequently observed (Figure 5). These results shed some light on the nature of the interaction between phytol and heptacosane with P-gp at the molecular level and would merit further mechanistic and kinetic studies. As for the data observed for the three inhibitors on the NBP site, verapamil can once again be considered a major inhibitor of ATPase activity. The calculated binding free energies show overlapping values between phytol and heptacosane, even though slightly lower than verapamil (Figure 6; Table 2). The mechanism of action of verapamil is a competitive inhibition in which substrate and inhibitor compete for the same binding site on the transporter and is based on the saturability of P-gp, for which an effective action requires a high dosage and consequently causes marked toxicity. Doxorubucin accumulation assays (Figure 7 and Figure 8; Table 3) confirmed that heptacosane inhibits efflux function of P-gp causing a significative accumulation of doxorubicin comparable to that caused by verapamil. Phytol does not have significative influence on the accumulation of doxorubicin. Doxorubicin intracellular accumulation in the presence of heptacosane strengthen its cytotoxic effect (Table 4). Docking data indicate a possible interaction of NBP site with phytol and heptacosane. Our results suggested that phytol and heptacosane act as a stimulator of ATP-dependent drug efflux transporter, acting as a substrate for transport by P-gp that stimulate P-gp ATPase activity, and lead to the inhibition of P-gp efflux function in the presence of another substrate such as verapamil (Figure 9). Interestingly, when used in combination there is no additive effect probably because they compete for the same binding site. P-gp inhibitors have proved to be unsatisfactory for the clinical use due to the inhibition of the physiological functionality of P-gp in healthy tissues, resulting in high toxicity which limits the application of these compounds. Our data indicate that phytol and heptacosane could be candidates as non-toxic inhibitors of ATPase activity and P-gp function. Our data indicate that phytol and heptacosane could be candidates as non-toxic inhibitors of ATPase activity and P-gp function. For these reasons, they could be used in combination with different anticancer chemotherapy drugs to improve their effectiveness in patients with MDR.

## 4. Materials and Methods

### 4.1. Cell Lines

HL-60 and HL-60R cells were cultured in Roswell Park Memorial Institute (RPMI) 1640. hTERT RPE-1 and 1-7HB2 cells were cultured in Dulbecco’s Modified Eagle Medium (DMEM) (HyClone Europe Ltd., Cramlington, UK), only for 1-7HB2 cells, supplemented with hydrocortisone (5 μg/mL) and insulin (10 μg/mL). All media were supplemented with 10% heat inactivated fetal calf serum, 2 mM L-glutamine, 100 units/mL penicillin and 100 µg/mL streptomycin (all reagents were from HyClone Europe Ltd., Cramling-ton, UK) in a humidified atmosphere at 37 °C in 5% CO_2_. HL-60 cells were obtained from ATCC^®^ (CCL-240, Rockville, MD, USA), while its variant HL-60R was selected for multidrug resistance (MDR) by exposure to gradually increasing concentrations of doxorubicin. The hTERT RPE-1 (ATCC^®^ CRL-4000TM) cells were kindly provided by Patrizia Cancemi (Department of Biological, Chemical and Pharmaceutical Science and Technology, University of Palermo, Italy). The 1-7HB2 (ECACC 10081201-Cancer Research Technology, London, UK) cells were kindly provided by Giulio Ghersi (Department of Biological, Chemical and Pharmaceutical Science and Technology, University of Palermo, Palermo, Italy).

### 4.2. Cell Growth Assays

Cells were seeded on 96-well plates at a density of 5000 cells/well and incubated overnight at 37 °C. After 24 h, at time 0 the medium was replaced with a fresh complete medium supplemented with heptacosane, phytol, verapamil, and doxorubicin (Sigma-Aldrich Srl, Milan, Italy), or their combinations at the indicated concentrations. After 72 h of treatment, 15 µL of Promega Corp. commercial solution (Madison, WI, USA) containing 3-(4,5-dimethylthiazol-2-yl)-5-(3-carboxymethoxyphenyl)-2-(4-sulfophenyl)-2H-tetrazolium (MTS) and phenazine ethosulfate was added to each well and the plates were incubated at 37 °C at 5% CO_2_ for 2 h. Using a microplate reader (iMark Microplate Reader; Bio-Rad Laboratories, Inc., Hercules, CA, USA), the bioreduction of the MTS dye was evaluated by measuring the absorbance of each well at 490 nm. Cytotoxicity was expressed as a percentage of measured absorbance relative to that of control cells.

Potentiation due to co-treatments were evaluated by viable cell count with trypan blue exclusion test. Data were expressed as mean ± standard error (S.E.) of at least three different experiments performed in duplicate.

### 4.3. NF-κB Activation

The DNA binding capacity of the NF-κB p65 subunit was evaluated in nuclear extracts obtained from HL-60R cells using the TransAM NF-κB and Nuclear ExtractTM kits (Active Motif, Carlsbad, CA, USA) according to manufacturer’s instructions. Results were reported as arbitrary units: one unit is the DNA binding capacity shown by 2.5 µg of whole cell extract from Jurkat cells stimulated with 12-otetradecanoylphorbol-13-acetate (TPA) + calcium ionophore (CI)/µg protein of HL-60R nuclear extracts.

### 4.4. Extraction of Cellular RNA and Reverse Transcription-Quantitative PCR (RT-qPCR)

Total RNA was extracted from cell lines using TRIzol reagent (Invitrogen Life Technologies, Carlsbad, CA, USA). For the evaluation of gene expression, RNA was reverse transcribed using a high-capacity complementary DNA (cDNA) reverse transcription kit (Applied Biosystems Life Technologies Inc., Foster City, CA, USA). The resulting cDNAs were subjected to real-time RT-PCR using the TaqMan Gene Expression Master Mix kit (Applied Biosystems Life Technologies Inc., Foster City, CA, USA) in triplicates. The PCR cycling conditions were as follows: denaturation at 50 °C for 2 min, annealing at 95 °C for 10 min, followed by 40 cycles of 95 °C for 15 s and extension at 60 °C for 60 min. The running of the samples and data collection were performed on a StepOne AB Real Time PCR system (Applied Biosystems Life Technologies Inc., Foster City, CA, USA). β-Actin was used as an internal standard. The specific TaqMan Assay used were: survivin Hs00153353, XIAP Hs00236913, Bcl-2 Hs00236329, and ABCB1 Hs00184005 (Applied Biosystems Life Technologies Inc., Foster City, CA, USA). Relative expression was calculated using the comparative Ct method [ΔCt = Ct (Target gene)—Ct (housekeeping gene)]. The Ct is the fractional cycle number at which the fluorescence of each sample passes the fixed threshold. Fluorescence was measured at 515–518 nm using StepOne AB Real Time PCR System software (Applied Biosystems Life Technologies Inc., Foster City, CA, USA). The ΔΔCt method for relative quantitation of gene expression was used to determine gene expression levels. The ΔΔCt was calculated by subtracting the ΔCt of the reference sample from the ΔCt of each sample. Fold change was generated using the equation 2^−ΔΔCt^.

### 4.5. Western Blotting Analysis

Wholecell lysates were obtained from HL-60R cells by lysis with RIPA buffer (Santa Cruz Biotechnology Inc., Dallas, TX, USA). A mass of 25 µg of proteins were separated using a 10% SDS-PAGE acrylamide gel. Nitrocellulose membrane transfer (Amersham, Pharmacia Biotech, Milan, Italy) was done using a semi-dry fast blot apparatus (Bio-Rad, Milan, Italy). After saturating the membranes with BSA 5% (*w*/*v*) in PBS-0.1% (*v*/*v*) Tween 20 for 1 h, the filters were incubated with primary antibodies against GAPDH (1: 20,000; Sigma-Aldrich Srl, Milan, Italy), survivin (1: 2000, Abcam Limited, Cambridge, UK), Bcl-2 (1:1000, Santa Cruz Biotechnology Inc., Santa Cruz, CA, USA), XIAP (1:500; Cell Signaling Technology, Inc. Danvers, MA, USA), and P-gp (1:100, Invitrogen, Milan, Italy). Using an advanced chemiluminescence detection kit, the SuperSignal West Femto Substrate of maximum sensitivity (Thermo Scientific Life Technologies Italia, Monza, Italy) and the Versa DOC imaging system (Laboratori BioRad, Milan, Italy) the hybridization was analyzed. Immunoblots were quantified by densitometry, normalized against GAPDH values and expressed as relative protein levels.

### 4.6. Active Site Prediction, Ligand Preparation, Docking, and Free Energy Calculation

The binding pocket of P-glycoprotein homology model has been searched by using SiteMap tool of Maestro Suite Software [56]. The Sitemap tool was used on the entire protein (all atoms in the workspace), setting at least 10 site points per receptor site and reporting up to 5 sites. The hydrophobicity parameter was set as more restrictive and a standard grid was set. The site maps identified were then cropped at 4 Å from the nearest points. The molecules were prepared using Schrödinger LigPrep v. 2018-4. The Force Field adopted was OPLS3, and Epik [57] was selected as ionization tool at pH 7.2 ± 0.2. The maximum number of conformers generated was set at 32. Docking study was performed using Glide docking tool, in extra precision (XP) using no constraints following a protocol previously reported [58]. Van der Waals radii were set at 0.8 and partial cutoff was 0.15 with flexible ligand sampling. Bias sampling torsion penalization for amides with non-planar conformation and Epik state penalties were added to the docking score. Lastly, MM-GBSA method for calculating the free energy of binding was performed with the MM-GBSA tool of Maestro 2020. VSGB solvation model was chosen using the OPLS3 Force Field as previously reported [59].

### 4.7. Determination of Doxorubicin Accumulation

The effects of heptacosane and phytol on the intracellular accumulation of doxorubicin were evaluated in the HL-60R cell line. The cells were seeded in 24-well plates at a density of 100,000/well cells and after 24 h, the cells were treated with heptacosane (20 or 50 µg/mL), phytol (25 or 50 µg/mL), with the combination of phytol (25 µg/mL) and heptacosane (50 µg/mL) or with verapamil (10 µM). After 24 h of incubation, doxorubicin, at 1 µg/mL, was added at different times: 30 min, 1 h, and 2 h. The cells were subsequently washed with PBS twice and then resuspended in a final volume of 400 µL of PBS. The fluorescence intensity of doxorubicin was measured by flow cytometry using a FACSAria III instrument (Becton Dickinson, Mountain View, CA, USA). The results are reported as a percentage of the fluorescence intensity relative to the control (mean ± S.E. of three experiments).

### 4.8. P-gp ATPase Activity Determination

P-gp ATPase activity was performed with Pgp-Glo™ Assay Systems (Promega, Madison, WI, USA) following manufacturer’s instructions. A range of concentrations of phytol, heptacosane, or their combination (test compound, TC) were added to a white 96-well plate in duplicate and incubated with recombinant human P-gp membranes. The compound sodium orthovanadate (Na_3_VO_4_, 0.25 mM) was used as the selective inhibitor of P-gp ATPase activity while negative control (no treatment, NT) with only Pgp-GLO assay buffer was used to provide a measure of unregulated ATPase activity. Verapamil (0.5 mM) is a P-gp substrate that stimulates P-gp ATPase activity and represents the positive control for drug stimulation of P-gp ATPase activity. The ATP standards curve serves to verify the correct execution of the assay. MgATP (5 mM) was added to initiate the ATPase activity; after 40 min of incubation at 37 °C, the reaction was stopped with 50 µL ATPase detection reagent and then incubated for 20 min at room temperature. Luminescence was measured using a GLOMAX Multidetection System (Promega). The data were presented as change in luminescence (ΔRLU), calculated as follows: ΔRLUbasal is the difference between the average luminescent signals from Na_3_VO_4_-treated samples (RLU Na_3_VO_4_) and untreated (NT) samples (RLUNT), ΔRLUTC that reflects P-gp ATPase activity in the presence of the test compounds, is the differ-ence between the average luminescent signals from Na_3_VO_4_-treated samples (RLU Na_3_VO_4_) and test compound-treated samples (RLUTC).

### 4.9. Statistical Analysis

The results are expressed as the average of three repetitions ± standard error. Statistical analysis was carried out by analysis of variance (one-way ANOVA) followed by Tukey’s test using Statistics ver. 12 (StatSoft Inc., Oklahoma City, OK, USA, 1984–2014).

## 5. Conclusions

**Figure 10:**
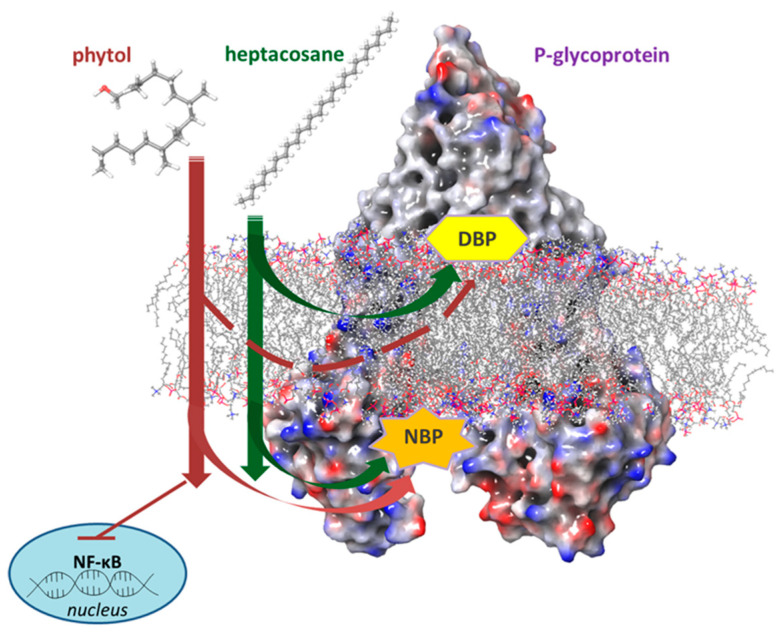
The potential mechanism of phytol and heptacosane on P-glycoprotein.

Phytol and heptacosane inhibit P-gp through different mechanisms. Phytol suppresses the expression of P-gp and acts as a substrate for P-gp transport and stimulators of the efflux transporter of ATP-dependent drugs without modifying the intracellular concentration of doxorubicin. Heptacosane acts as a substrate and potent P-gp inhibitor, demonstrating the ability to retain the substrate doxorubin inside the cell and enhancing its cytotoxic effects. Heptacosane has the potential to be used in combination with chemotherapy drugs (Figure 10). We demonstrated that in a tumor model with MDR phenotype, these non-toxic compounds can be used also at high concentration to revert P-gp mediated multidrug resistance. 

## Figures and Tables

**Table 1:** IC_50_ values of the three cell lines treated with phytol and heptacosane.

Treatment	HL-60	HL-60R	hTERT RPE-1	1-7HB2
	IC_50_	IC_50_	IC_50_	IC_50_
**phytol**	>100 µg/mL	>100 µg/mL	>100 µg/mL	>100 µg/mL
**heptacosane**	>100 µg/mL	>100 µg/mL	>100 µg/mL	>100 µg/mL

**Table 2:** Binding free energy, type of interaction and involved key residues for verapamil, doxorubicin, heptacosane and phytol in the predicted binding sites of P-gp.

	Compounds	Type of Interaction	Key Residue	Binding Free Energy (Kcal/mol)
**DBP**	**doxorubicin**	H-Bond H-Bond Pi-stacking	Gln725 Gln990 Phe732	−52.1
	**verapamil**	H-Bond Hydrophobic	Gln725 Phe72, Tyr307, Phe336, Phe728, Ala729, Phe732, Phe759, Cys956, Tyr953, Phe957, Val982, Phe978, Phe983	−82.7
	**heptacosane**	Hydrophobic	Phe72, Phe335, Phe336, Leu339, Ile340, Phe343, Phe732, Leu861, Cys956, Tyr953, Ile868, Phe957, Phe978, Val982	−114.2
	**phytol**	2 H-Bonds Hydrophobic	Gln725 Tyr310, Phe335, Phe336, Ile340, Phe343, Phe728, Ala729, Phe732, Phe759, Phe983	−75.8
**NBP**	**verapamil**	Pi-stacking	Tyr1044	−42.3
	**heptacosane**	Hydrophobic	Asp 800, Val801, Ser802, Phe804, Asp805	−30.2
	**phytol**	H-bond	Ser1117	−29.7

**Table 3:** Effects of phytol and heptacosane on intracellular accumulation of doxorubicin in HL-60 and HL-60R cell lines. The cells were treated with phytol, heptacosane (hepta) alone and in combination, or verapamil. After 24 h of incubation, doxorubicin (doxo) was added at different times. The intracellular accumulation of doxorubicin was measured by flow cytometric analysis. The results are presented as percentage of fluorescence intensity (means ± standard error of three experiments).

	Fluorescence %	
Treatment	HL-60	HL-60R
Control	0.05 ± 0.03	0.05 ± 0.03
doxo 1 µg/mL 30 min	98.4 ± 0.42 *	7.85 ± 0.10 *
doxo 1 µg/mL 1 h	99.9 ± 0.07 *	9.80 ± 0.14 *
doxo 1 µg/mL 2 h	100 ± 0.0 *	21.8 ± 0.14 *
phytol 25 µg/mL	0.05 ± 0.03	0.05 ± 0.03
heptacosane 50 µg/mL	0.15 ± 0.03	0.25 ± 0.03
phytol 25 µg/mL + heptacosane 50 µg/mL	0.15 ± 0.03	0.05 ± 0.03
verapamil 10 µM	0.05 ± 0.02	0.05 ± 0.03
phytol 25 µg/mL + doxo 1 µg/mL 30 min	99.7 ± 0.12 *	4.70 ± 0.20 *
phytol 25 µg/mL + doxo 1 µg/mL 1 h	99.9 ± 0.03 *	10.8 ± 0.90 *
phytol 25 µg/mL + doxo 1 µg/mL 2 h	100 ± 0.0 *	17.1 ± 1.80 *
heptacosane 50 µg/mL + doxo 1 µg/mL 30 min	98.9 ± 0.67 *	31.0 ± 0.70 *^,a^
heptacosane 50 µg/mL + doxo 1 µg/mL L h	100 ± 0.0 *	49.6 ± 0.25 *^,a^
heptacosane 50 µg/mL + doxo 1 µg/mL 2 h	100 ± 0.0 *	71.7 ± 0.12 *^,a^
phytol 25 µg/mL + hepta 50 µg/mL + doxo 1 µg/mL 30 min	99.9 ± 0.03 *	8.35 ± 0.10 *
phytol 25 µg/mL + hepta 50 µg/mL + doxo 1 µg/mL 1 h	100 ± 0.0 *	19.6 ± 0.30 *^,a^
phytol 25 µg/mL + hepta 50 µg/mL + doxo 1 µg/mL 2 h	100 ± 0.0 *	39.2 ± 0.57 *^,a^
verapamil 10 µM + doxo 1 µg/mL 30 min	99.5 ± 0.35 *	58.7 ± 0.53 *^,a^
verapamil 10 µM + doxo 1 µg/mL 1 h	99.0 ± 0.70 *	70.8 ± 0.57 *^,a^
verapamil 10 µM + doxo 1 µg/mL 2 h	100 ± 0.0 *	98.0 ± 0.70 *^,a^

* *p* < 0.01, vs. the control. ^a^ *p* < 0.01 represent significant differences among the treatments in combination with phytol or heptacosane or verapamil compared to doxorubicin alone for the same time (one-way ANOVA followed by Tukey’s test).

**Table 4:** Results of cell counting analysis in HL-60R cells following 48 h of treatment with heptacosane (hepta) and doxorubicin (doxo), either alone or in combination.

Treatments	Cell Growth Inhibition, %	Expected, %
heptacosane 20 µg/mL	9.0 ± 0.7	
heptacosane 50 µg/mL	17.0 ± 0.7 *	
doxo 0.25 µg/mL	10.5 ± 3.9	
doxo 0.5 µg/mL	17.5 ± 1.8 *	
hepta 20 µg/mL + doxo 0.25 µg/mL	22.5 ± 5.3 **	18.7 ± 3.0 *
hepta 20 µg/mL + doxo 0.5 µg/mL	48.5 ± 0.3 **	27.0 ± 1.0 **^,b^
hepta 50 µg/mL + doxo 0.25 µg/mL	50.5 ± 2.5 **	25.5 ± 3.9 **
hepta 50 µg/mL + doxo 0.5 µg/mL	65.5 ± 0.3 **	31.5 ± 2.0 **^,a^

Data are expressed as the mean ± standard error of three independent experiments. ** *p* < 0.01, * *p* < 0.05, vs. the control. ^a^ *p* < 0.01, ^b^ *p* < 0.05, expected vs. observed (one-way ANOVA followed by Tukey’s test).

## Data Availability

Data is contained within the article and Supplementary Materials.

## Supplementary Materials

The following supporting information can be downloaded at: https://www.mdpi.com/article/10.3390/ph15030356/s1, Figure S1: Ramachandran plot of the obtained homology model for P-gp. Refs. [60,61].
