# Supplementary material for: Phytol and Heptacosane Are Possible Tools to Overcome Multidrug Resistance in an In Vitro Model of Acute Myeloid Leukemia
Source: Pharmaceuticals (Basel). 2022 Mar 15;15(3):356. doi: 10.3390/ph15030356 (PMC8952646; doi:10.3390/ph15030356)
Supplement: Supplementary file 1 [file pharmaceuticals-15-00356-s001.zip › pharmaceuticals-1621430-supplementary.pdf]

Supplementary material

Materials and methods

Homology modeling and protein preparation

Starting from the MDR1_HUMAN sequence P08183 (https://www.uniprot.org), the homology model structure was modeled in the SWISS-MODEL workspace (swissmodel.expasy.org/workspace). The PDB ID 4Q9K was used as a template for the apoprotein. The model obtained was refined with the Protein Preparation Wizard tool of Maestro Suite Software in order to optimize the protein structure improprieties, such as correct assignment of connection bonds, addition of missing hydrogens, and analysis of atomic clashes. PROPKA was used to check for the protonation state of ionizable protein groups at pH = 7.0. Protein was refined using restrained minimization with OPLS3 as Force Field. The model obtained was analyzed by RAMPAGE (http://mordred.bioc.cam.ac.uk/~rapper/rampage.php). A Ramachandran Plot (measuring the side-chains torsion angles) examined the quality of the protein structure. Remarkably, the 94.5% of the residues of our homology model fell in the most favored regions, 4.8% in allowed regions and just 0.7% in outlier regions. Additionally, the global and perresidue model quality has been assessed using the QMEAN scoring function (QMEAN value = -2.56).

Results

Homology Modeling

The protein sequence of P-glycoprotein (MDR1_HUMAN sequence P08183) was downloaded from the UNIPROT database (https://www.uniprot.org). Then, the SWISS-MODEL template library was searched with BLAST [1] and HHBlits [2] for evolutionary related structures matching the target sequence. According to the HHblits search result and overall 50 templates, the M*us musculus* P-glycoprotein (PDB ID: 4Q9K) give the most accurate homology model (Figure 4). The quality of the selected model was validated by means of the Ramachandran plot and the QMEAN score. The Ramachandran plot is a graphical representation of the backbone conformation of each residue in a protein. As reported in Figure S1, the plot suggested that 94.4% of the residues occupy the blue regions (favored regions), 4.8% of the residues occupy the light blue regions (allowed regions) and just 0.7% of the residues occupy the outlier regions. Finally, the QMEAN score value (-2.56) is a confirmation of the goodness of the model. The homology model was used for further analysis.

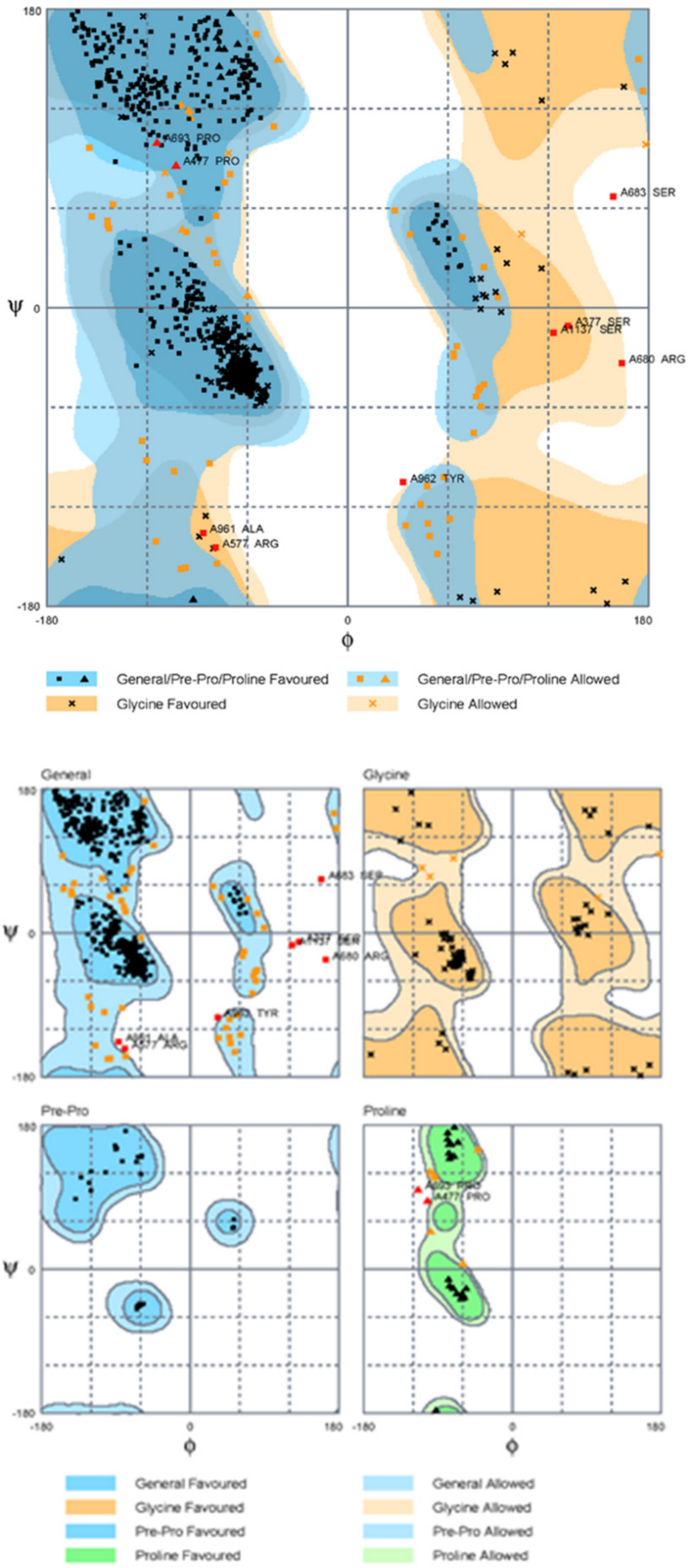

Figure S1. Ramachandran plot of the obtained homology model for P-gp.

References

1. Camacho, C.; Coulouris, G.; Avagyan, V.; Ma, N.; Papadopoulos, J.; Bealer, K.; Madden, T.L. BLAST+: Architecture and applications. BMC Bioinform. 2009, 10, 421–430.

2. Remmert, M.; Biegert, A.; Hauser, A.; Söding, J.H. Hblits: Lightning-fast iterative protein sequence searching by HMM-HMM alignment. Nat. Methods 2011, 9, 173–175.
